# Perceptions of Burden and Preparedness for Caregiving among the Family Caregivers of Hospitalised Older Adults: A Cross-Sectional Study

**DOI:** 10.3390/geriatrics7010019

**Published:** 2022-02-15

**Authors:** Carla Gomes da Rocha, Béatrice Perrenoud, Anne-Sylvie Ramelet

**Affiliations:** 1 Acute Geriatric Care Unit, Lausanne University Hospital, Avenue Pierre Decker 5, CH-1011 Lausanne, Switzerland; 2 Nursing Directorate, Lausanne University Hospital, Rue du Bugnon 21, CH-1011 Lausanne, Switzerland; beatrice.perrenoud@chuv.ch; 3 La Source Institute and School of Nursing, University of Applied Sciences and Arts Western Switzerland, Avenue Vinet 30, CH-1004 Lausanne, Switzerland; 4 Institute of Higher Education and Research in Healthcare, University of Lausanne, Biopôle 2, Route de la Corniche 10, CH-1010 Lausanne, Switzerland; anne-sylvie.ramelet@chuv.ch

**Keywords:** burden, caregivers, older adults, perception, preparedness

## Abstract

Background: Due to the increasing care needs of older adults, family caregivers are more and more solicited. This can have a negative impact on their quality of life related to a lack of preparedness for caregiving and feelings of burden. Objectives: To measure perceptions of burden and preparedness for caregiving among the family caregivers of hospitalised older adults, and to explore their possible associations. Methods: A cross-sectional study conducted in two university hospital geriatrics wards in Switzerland. Principal family caregivers of hospitalised older adults were invited to complete sociodemographic, the Zarit Burden Interview, and the Preparedness for Caregiving Scale questionnaires. Descriptive and correlational data analyses were performed. Results: Of the 38 responding caregivers, 80% provided informal care to their spouse or parent; 45% reported a lack of preparedness to provide care and 61% reported substantial levels of burden. There was no statistically significant correlation between preparedness and burden (*ρ* ≤ −0.30, *p* = 0.07). Conclusions: A significant proportion of caregivers reported burden and a lack of preparedness. Healthcare professionals should provide adequate support to help informal caregivers to fulfil their roles.

## 1. Introduction

By 2050, there are expected to be two billion people over 60 years old on the planet compared with 900 million in 2015 [1], leading to concurrent increases in the prevalence of chronic diseases and demands for primary and long-term care [2]. Around the world, family members are the main caregiving resource for dependent community-dwelling older adults, and demand for informal care (unremunerated care provided by family members or significant others) is growing [3,4,5]. However, most current formal healthcare services around the world are insufficiently prepared for supporting family caregivers, and this is challenging for nurses and other healthcare professionals [6,7,8,9]. Indeed, they may have difficulty identifying those family caregivers who are struggling, and thus have difficulties implementing the comprehensive nursing interventions essential to maintaining their health and well-being [10]. When inadequately prepared and supported, family caregivers can experience such significant burden that their own quality of life (QoL) is greatly affected [11].

The concept of burden appears to be directly related to informal care [8], and it includes the physical, psychological, emotional, social, and financial problems experienced by caregivers [12]. It is relevant to distinguish between subjective and objective burdens: subjective burden is defined as the emotional impact that an older person’s presence and behaviour may have, being directly linked to the caregiver’s feelings of shame and embarrassment (e.g., a decreased willingness to invite friends to the house because of the older person’s presence); objective burden is defined as disruptions to the family caregiver’s everyday life, equally triggered by the older person’s presence (e.g., financial burden, changes in daily routines like meal times and sleeping times, new supervisory tasks, among others) [13,14]. Researchers working with the Zarit Burden Interview refer to three dimensions of burden: the *overall burden* (referring to the overall dimension of the burden experienced by the subject), *personal strain* (referring to how stressful the experience is from a personal perspective), and *role strain* (referring to the stress resulting from role conflict or overload) [14,15,16,17]. Burden tends to increase considerably over time, potentially causing deteriorations to well-being and QoL among family caregivers [18,19]. A high prevalence (85%) of burden among family caregivers has been reported previously, with 60% suffering a mild-to-moderate burden and 23% suffering a moderate-to-severe burden [20].

The predictive factors of burden are related to both the older adult and the family caregiver. Those related to older adults are functional decline (difficulty in performing the basic activities of daily living, or ADLs, or the instrumental activities of daily living, or IADLs), being head of the household, being married, a low educational level, depressive symptomatology, sleep disorders, cognitive disorders, and the presence of behavioural and psychological symptoms related to dementia [21,22,23,24,25,26]. Those related to family caregivers include spending > 40 h per week giving informal care, providing assistance with a high number of ADLs and IADLs, using inefficient coping strategies, being married, cohabitation with the older relative, and a low educational level [21,27,28,29,30,31]. Several studies have reported higher burden among female family caregivers [15,18,24,27,31,32]. However, variables associated with the older adult seem to affect the family caregiver’s burden the most [33]. Caregivers’ perceptions of their own health status as poor have been associated with burden, depression, and anxiety [29,34,35]. Furthermore, according to some studies, perceptions of burden seem to predict depression and anxiety among family caregivers [15,36].

The concept of preparedness for caregiving is defined as the caregivers’ subjective perceptions of feeling prepared to assist a family member or loved one in an informal care setting, such as providing physical care, emotional support, organising in-home support services, and stress management [37,38]. Assessing a caregiver’s preparedness level is important for identifying those at risk of having a lower QoL and being overburdened [37]. Although some studies have shown that most caregivers felt well prepared to assist [37,39], others have shown that a significant proportion of them (close to a third) reported low preparedness levels [40]. Cultural variances may help explain these differences. Preparedness also seems to fluctuate over time, with a tendency towards feeling more prepared when starting to provide informal care [19].

Research has revealed an inverse association between preparedness and burden: higher preparedness levels were associated with the lowest levels of burden [41]. Another study associated lower preparedness levels with higher caregiver burden among a group of high-intensity caregivers [34]. Among family caregivers, low-to-moderate preparedness levels have been associated with poor mental health and high levels of demand during their caregiving [34,42]. In contrast, a high preparedness level was associated with better mental health [37,43]. Social support, the location of care, time since diagnosis, and patient age were not associated with preparedness [43]. In summary, higher preparedness levels seem to be related to lower levels of anxiety and burden and, as a result, better mental health.

To the best of our knowledge, there has been no recently published research exploring the associations between preparedness and the burden faced by family caregivers of older adult populations. In the rapidly ageing and changing context of care, this gap in the knowledge needs to be addressed. This study thus aimed to describe perceived burden and preparedness for caregiving among the family caregivers of hospitalised older adults.

The study’s objectives were: (I) to describe family caregivers’ perceived preparedness for caregiving and its possible associations with patients’ sociodemographic and clinical characteristics; (II) to describe family caregivers’ perceived burden and its possible associations with patients’ sociodemographic and clinical characteristics; and (III) to identify possible associations between perceived preparedness for caregiving and perceived burden.

## 2. Materials and Methods

This study was completed with reference to the *Strengthening the Reporting of Observational Studies in Epidemiology* (STROBE) *Statement* [44].

### 2.1. Study Design, Setting, and Sample

This cross-sectional study was conducted in two university hospital geriatrics wards (one acute care and one rehabilitation) in Western Switzerland. Based on the exploratory nature of the study, the *n* = 30 rule of thumb for convenience samples, the number of patients admitted per month, an estimated participation rate of 50%, and the time allocated for data collection, we aimed to recruit a convenience sample of 40 dyads of hospitalised older adults and their main family caregivers.

Older adult inclusion criteria were being hospitalised in one of the units where the study was taking place, being 65 years old or more, and understanding, reading, and writing French fluently. Older adult exclusion criteria were an unstable clinical situation, being in an end-of-life care situation, and being institutionalised. If the older adult was unable to identify their primary family caregiver (e.g., due to cognitive impairment), their legal representative was contacted to decide whether they could participate in the dyad study.

Family caregiver inclusion criteria were having been the patient’s primary caregiver for at least six months, understanding, reading, and writing French fluently, being 18 years old or more, and having been identified as the primary caregiver by the patient themself or by their legal representative. The family caregiver exclusion criterion was being both the family caregiver and the patient’s legal representative.

### 2.2. Recruitment Procedure

Following approval by the local Ethics Committee, participant recruitment and data collection took place between October 2015 and April 2016. The first step of the recruitment phase (*identification of family caregivers* via *patients*) was carried out by the main researcher (C.G.d.R.) in collaboration with nursing staff (the head nurse or the patient’s primary nurse), who identified eligible patients each time a new patient was admitted to the ward. The main researcher visited the units about three times a week to be informed about potential new participants previously identified by nursing staff. After screening, eligible patients were approached by the main researcher for consent to collect their sociodemographic and clinical data from their medical charts, and to invite their family caregivers to participate.

The main researcher recruited family caregivers via telephone calls or face-to-face meetings. Consenting family caregivers subsequently completed a questionnaire either handed directly to them or sent by post.

### 2.3. Measures

Patients’ sociodemographic and clinical data were collected from their medical charts by the main researcher. Family caregivers’ sociodemographics, and preparedness- and burden-related data were collected using self-administered questionnaires. Caregivers’ perceived preparedness levels were measured using the eight-item Preparedness for Caregiving Scale (PCS), translated into French according to Wild’s methodology [38,45]. Responses were scored using a 5-point Likert scale ranging from 0 (“not at all prepared”) to 4 (“very well prepared”). The final PCS scores were obtained by calculating the mean score across the eight items which varied between 0 and 4, with a high score indicating a high preparedness level. The dimensions of the PCS include preparedness for physical care, emotional support, organising in-home support services, and stress management [19,41]. The PCS has moderate-to-high reported internal consistency (Cronbach’s alpha 0.88–0.93) [46,47]. The original study demonstrated the instrument’s construct and content validity from the level of caregivers’ concern (Cronbach’s alpha 0.84) to their lack of resources (Cronbach’s alpha 0.77) [38,48].

Perception of burden was measured using the 22-item version of the Zarit Burden Interview (ZBI) [49,50]. Responses were scored using a 5-point Likert scale ranging from 0 (“never”) to 4 (“almost always”). The final ZBI score was obtained by summing the 22 item scores and ranged from 0 to 88, with higher scores indicating a higher perception of burden [51]. The dimensions of burden retained for the present study were overall burden, personal strain, and role strain [14,15].

The ZBI’s French version showed an internal consistency (Cronbach’s alpha) of 0.85 and a Spearman–Brown coefficient of 0.87 [14].

### 2.4. Statistical Analysis

We performed descriptive statistics to present participants’ sociodemographic and clinical characteristics, and to describe the levels of preparedness for caregiving and family caregivers’ burden. The parametric properties of the PCS and ZBI scores were analysed for the normality of their distributions using the Shapiro–Wilk test. Given the small size of the sample (*n* = 38), bivariate analyses between the PCS and ZBI scores (treated as continuous variables) associated with the sociodemographic and clinical characteristics of family caregivers and their older relatives (treated as dichotomous variables) were performed using the Kruskal–Wallis Test and Fisher’s Exact Test. The Kruskal–Wallis test was preferred because either the distribution was asymmetric or the variances were not homogeneous. The PCS and ZBI scores were processed continuously by dichotomising the scores at their medians according to their categories (mild/moderate/high for the ZBI; five possible levels of preparedness for the PCS). Spearman’s nonparametric correlation coefficient (Rho) was used to test for correlations between the dimensions of burden and preparedness for caregiving (asymmetric distributions). Statistical significance was set at *p* < 0.05, with a 95% confidence interval. Statistical analysis was performed using Stata 14 software (StataCorp. 2015. Stata Statistical Software: Release 14. StataCorp LP, College Station, TX, USA).

## 3. Results

**Figure 1:**
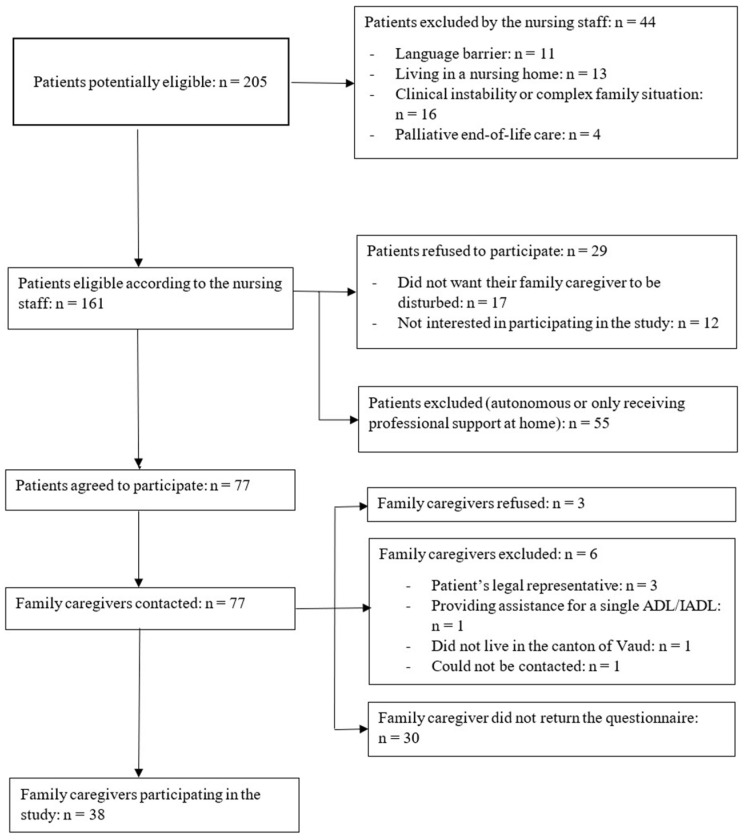
Flow diagram.

A total of 38 family caregivers and 38 older adults participated in the study (Figure 1).

### 3.1. Hospitalised Older Adults’ Characteristics

The mean patient age was 84 (*SD* = 6.93) years old, most were female (55%, *n* = 21), and the majority were either widowed (47%, *n* = 18) or married (45%, *n* = 17); half lived alone (50%, *n* = 19), and the other half lived with a family member. Eighty-one per cent (*n* = 30) lived in urban areas.

Regarding professional home care before hospitalisation, older adults had mostly been assisted with bathing (71%, *n* = 27), dressing and undressing (34%, *n* = 13), housekeeping, laundry and shopping (16%; *n* = 6), and food preparation and delivery (16%, *n* = 6).

Their most frequent health problems were mental and behavioural disorders (47%, *n* = 18), falls (34%, *n* = 13), gait and balance disorders (32%, *n* = 12), circulatory system diseases (26%, *n* = 10), and osteoarticular system, muscle, and connective tissue diseases (21%; *n* = 8). In 40% of the sample (*n* = 15), at least two medical diagnoses were documented (*Mdn* = 2, *Min* = 1, *Max* = 6).

The most frequent comorbidities included circulatory system diseases (76%, *n* = 29), endocrine, nutritional, and metabolic diseases (53%, *n* = 20), genitourinary system diseases (50%; *n* = 19), mental and behavioural disorders (47%, *n* = 18), osteoarticular system, muscle, and connective tissue diseases (45%, *n* = 17), and gait and balance disorders (45%, *n* = 17). Almost half (47%, *n* = 18) had four to five comorbidities (*Mdn* = 4, *Min* = 2, *Max* = 10).

A total of 11 (29%) hospitalised older adults were independent in their ADLs at home, with 27 (71%) being partially dependent and none being completely dependent. Only one older adult (3%) carried out all their IADLs independently at home, whereas 82% (*n* = 31) were partially dependent and 16% (*n* = 6) were completely dependent. Overall, the older adults were dependent on assistance with two ADLs and six IADLs (median scores) before their hospitalization, according to the Katz Index [52,53] and the Lawton Scale.

The present results revealed that 69% (*n* = 24) of our older adults returned home after their hospital discharge, whereas 31% (*n* = 11) were institutionalised in nursing homes.

### 3.2. Family Caregivers’ Characteristics

Family caregivers’ characteristics are shown in Table 1 and Table 2.

Family caregivers reported spending an average of 32 h a week on informal care, for between one and four years for 53% of them (*n* = 18). More than half (55%, *n* = 21) reported the existence of at least one other informal caregiver in their situation. Many caregivers were satisfied with the professional home care services received by their older relatives (37%, *n* = 10). Almost two-thirds of caregivers reported that they had a good health status (61%, *n* = 23), whereas 26% (*n* = 10) felt that they had an average health status.

### 3.3. Family Caregivers’ Perception Levels of Preparedness for Caregiving

The study’s mean PCS score was 1.76 (0.91), indicating that overall, family caregivers felt “not too well prepared” to assist their older relatives. Fewer than half (42%, *n* = 16) of family caregivers felt “not too well prepared”, and 37% (*n* = 14) felt “somewhat well prepared” to offer that assistance (Table 3).

Regarding “preparedness to provide physical care assistance”, 35% (*n* = 13) of family caregivers reported being either “not at all prepared” or “not too well prepared”, whereas 66% (*n* = 25) reported being “somewhat” or “very well prepared”. Concerning “preparedness to offer emotional support”, 32% (*n* = 12) of family caregivers reported low preparedness levels and 69% (*n* = 26) reported high preparedness levels. Regarding “preparedness for organising in-home support services”, 26% (*n* = 10) of family caregivers perceived their preparedness to be low, whereas 74% (*n* = 28) reported high preparedness levels. Concerning “preparedness to manage stress”, 34% (*n* = 13) of relatives reported feeling “not too well prepared”, whereas 66% (*n* = 25) reported sufficiently high preparedness levels.

### 3.4. Associations between Preparedness for Caregiving and Older Adults’ Characteristics

The total PCS score and the length of time that family caregivers had been providing informal care showed no statistically significant association (*M* = 1.82, *SD* = 0.93, *p* > 0.05). On average, family caregivers who had provided informal care for fewer than five years felt “not too well prepared” to assist (*M* = 1.55, *SD* = 0.92). Those who had provided informal care for five years or more felt “somewhat well prepared” (*M* = 2.12, *SD* = 0.88). Other family caregivers’ sociodemographic variables, such as gender, residential area, employment status, educational level, and cohabitation with the older relative, were not significantly associated with PCS scores (*p* > 0.05).

Older adults’ age did not show any statistically significant association with PCS scores, and among comorbidities, mental and behavioural disorders were the only category that did significantly influence it (*M* = 1.76, *SD* = 0.91, *p* = 0.04). Family caregivers dealing with mental and behavioural disorders reported lower preparedness levels for caregiving (a PCS score of 1.4, or “not too well prepared”). Their perceptions of preparedness for caregiving were better when they assisted older relatives who had not been diagnosed with mental and behavioural disorders (*M* = 2.05, *SD* = 0.94), where they felt “somewhat well prepared”.

### 3.5. Family Caregivers’ Perception of Burden

Family caregivers’ mean score for overall perceived burden was 24.32 (*SD* = 15.04), or “moderate burden” overall. More precisely, 40% (*n* = 15) of them perceived a “mild burden”, 42% (*n* = 16) reported a “moderate burden”, and 18% (*n* = 7) perceived a “high burden”. Overall, no participants expressed a level of “severe burden”.

Concerning the “personal strain” and “role strain” dimensions of burden, 42% (*n* = 16) of family caregivers reported high levels of these types of burden, and 24% (*n* = 9) described severe burden.

The analysis of the total ZBI scores indicated a statistically significant association between the mean levels of burden and family caregivers who were themselves receiving professional home care services (*n* = 4) (*M* = 24.72, *SD* = 15.02, *p* = 0.02). The results showed a moderate level of burden (*M* = 26.5, *SD* = 14.7) among those caregivers who were not themselves receiving support from professional home care services (*n* = 33).

Among family caregivers perceiving a considerable burden (above the median score of 24), 65% (*n* = 13) were under 66.5 years old.

### 3.6. Associations between Family Caregivers’ Perceptions of Burden and the Sociodemographic and Clinical Characteristics of Their Older Adult Relatives

The association between ZBI scores and the “diseases of the nervous system” comorbidity category was statistically significant (*M* = 24.31, *SD* = 15.04, *p* = 0.02). The mean ZBI score was higher among caregivers who assisted people with this category of comorbidity than those who did not (*M* = 33.4 vs. *M* = 21.1, *p* = 0.02).

Perception of burden was also associated with comorbidities such as osteoarticular system, muscle, and connective tissue diseases (*M* = 24.31, *SD* = 15.04, *p* = 0.01). If the older adult relative was affected by this type of comorbidity, the ZBI score was higher (*M* = 31.4), indicating a moderate burden (vs. *M* = 18.6, indicating a mild burden, if the older adult did not suffer from this type of comorbidity).

The association between the median ZBI score (*Mdn* = 24) and the category of osteoarticular system, muscle, and connective tissue diseases was also statistically significant (*p* = 0.01), indicating that 76.5% (*n* = 13) of caregivers felt a moderate or high level of burden (above the median score). On the other hand, in the absence of this type of comorbidities, 66.7% (*n* = 14) of the family caregivers were below the median score.

### 3.7. Associations between the Dimensions of Preparedness for Caregiving and the Dimensions of Burden

Correlation coefficients indicated that the dimensions of the Preparedness for Caregiving Scale were very weakly negatively correlated with the dimensions of the ZBI. However, three of these associations were slightly different (ρ > −0.30, *p* < 0.05): (a) the PCS dimension of emotional support and the ZBI dimension of role strain; (b) the PCS dimension of organising in-home support services and the ZBI dimension of role strain; and (c) the PCS dimension of overall preparedness and the ZBI dimension of role strain (Table 4).

## 4. Discussion

### 4.1. Older Adults’ Sociodemographic and Clinical Characteristics

The present study involved more older aged adults than in previous international studies [21,23,28,42,54]. By international comparison, Swiss people have a very favourable life expectancy at birth (81 years for men; 85.2 years for women), which could explain this difference [55]. Older patients’ marital statuses were consistent with what has been reported in previous research [21,23,24,34]. The findings regarding their living situation, however, did not match those from Turkish [21] and Taiwanese [42] studies, where 87% and 96% of older adults lived with a family member, respectively. These differences might be explained by cultural specificities, as well as by the levels of development and effectiveness of home care services networks. These services are quite developed in Switzerland, helping to keep older adults at home for as long as possible, even if they live alone [56]. A Belgian study showed that 40% of older adults lived with family members [24], which seems more consistent with the present findings in a more similar cultural and socioeconomic context.

The present study’s results emphasized the importance of considering potential explanatory factors of functional dependence. Some research has focused on people with dementia [42,57], and our study suggested that participants’ most frequent health problems were mental and behavioural disorders. Problems involving gait, balance disorders, or falls were also significantly represented in our sample, and almost half of the older adults had four or more active comorbidities involving vital organs, suggesting the need for complex care and a high risk of having a frailty syndrome [58]. Some degree of complexity is common when treating chronic diseases in polymorbid older adults [58]. However, and regardless of the impact on their functional health, most of our study’s older adults returned home after hospital discharge. Indeed, in national comparisons, the canton where the study was conducted has one of the lowest rates of institutionalisation in Switzerland [59].

### 4.2. Sociodemographic Characteristics of Family Caregivers

The typical profile of the caregiver assisting the older adults hospitalised in our study’s geriatrics wards (a retired, married woman with a high educational level and a low-to-midrange monthly income, caring for her spouse or parent) was consistent with those presented in previous research [23,24,29,33,34,60], except for employment status and monthly income. According to the United Nations, older adults are at particular risk of poverty [61]. Although the consulted literature did not address monthly income, our results suggested that income was indeed a concern for one-fifth of the participants, and finances are not a minor issue when discussing family caregivers’ burden. Another aspect worth highlighting is the average family caregiver’s age: at 66 years old they can often be considered an older adult themselves. Of course, the age at which an individual becomes ‘old’ is debatable, depending on different perspectives and who is making the analysis—this is indeed a complex topic [62]. In any case, from a biological point of view, the process of ageing leads to a progressive reduction in physical and mental capacities, and to an increased risk of disease. These changes are not linear, nor are they exclusively associated with an individual’s age in years [63]. However, they may point to a certain vulnerability and should be considered by health professionals when interacting with an ageing family caregiver. Being retired (another characteristic of the typical family caregiver), possibly recently, may also mean that the person is going through a major life transition themselves [64], and is thus more vulnerable to certain phenomena, including burden.

### 4.3. Family Caregivers’ Perceptions of Their Level of Preparedness for Caregiving

The study’s average total PCS score was low. Most family caregivers reported themselves as “not too well prepared” to begin providing assistance. These results diverge from those of other studies [34,37,39,40], where caregivers reported relatively high preparedness levels. However, in one North American study [19], the authors highlighted that caregivers’ perceived preparedness was high at baseline, but decreased over time. These findings appear to be consistent with our study because the informal caregivers had been providing care for at least one year—some up to 10 years. Regarding the dimensions of the PCS (physical care, emotional support, organising in-home support services, and stress management), most caregivers had reported satisfactory preparedness levels. The consulted literature did not directly address these issues, focusing on the overall PCS score instead. Nevertheless, some papers have documented very low use of home care support services available to family caregivers [54,65], and this seems to diverge from the current study’s results in terms of the perceived preparedness to organise in-home support services. Some authors have noted that caregivers seemed to wait for their burden to become too great before turning to support services [65]. Perhaps the hospitalisation of older relatives is also a way of supporting the organisation or optimisation of these services, through the intervention of nurses or other professionals.

Among older adults, mental and behavioural disorders formed the only category of comorbidities that significantly influenced preparedness for caregiving. This finding was consistent with the results from two other studies [31,42], and it indicated better preparedness for caregiving when the older person did not present these types of pathologies. A previous study has associated lower levels of preparedness with poorer mental health and high levels of demand for caregiving [42].

The relatively low PCS scores of the family caregivers in our sample could be assumed to be a low baseline preparedness level that would improve over time. In that case, however, if the association with “preparedness for caregiving” is inversely proportional, the burden on caregivers should decrease over the years [41], but this is inconsistent with the tendency for burden to increase over time [18,19]. Indeed, it seems that family caregivers feel better prepared as the years pass, thanks to their experiences, learning, and better knowledge of the support they might expect from the healthcare network: the perception of being properly prepared reduces feelings of burden. The quality of the support provided by healthcare networks tends to maintain perceptions of high preparedness.

Our study did not show any statistically significant associations between high levels of preparedness and the caregiver’s gender, the age of the older person, or cohabitation of the dyads, as suggested in previous research [43].

### 4.4. Family Caregivers’ Perceived Levels of Burden

The present results showed a moderate or high prevalence of burden among almost two-thirds of participants—twice as high as found in an earlier study [12]. Demographic ageing and the increasing complexity of care (including informal care) may explain why family caregivers currently perceive a higher burden. In contexts involving pathological ageing, difficulties in carrying out the ADLs and IADLs, plus the presence of sleep disorders, affective disorders, and the behavioural and psychological symptoms of dementia, this can predict a caregiver’s burden [15,20,22,26,31,33]. Indeed, the present study’s older adults were functionally dependent in two ADLs and six IADLs (median scores); they also frequently had mental and behavioural disorders as their primary diagnosis or as a comorbidity. Concerning the factors predicting burden associated with caregivers (>40 h of informal care per week, assistance in accomplishing a high number of ADLs and IADLs, being married, cohabitation with the older adult patient, and being female), the present study’s results were in line with previous research [18,20,36,42,66,67]. Family caregivers provided an average of 32 h of informal care per week, not far from the 40 h cut-off, assisting in a considerable number of ADLs and IADLs. Approximately 40% of them were married and cohabited with the older person, and about 80% were women. This evidence emphasizes the benefits of examining burden in this study. The total mean perceived burden score indicated “moderate burden” among family caregivers, partly because none of the participants reported severe levels of burden, pushing the total mean score downwards. This may be explained by the older adult’s hospitalisation, a period during which the caregiver probably felt less taxed by their normal informal care activities, thus reporting a lower perceived level of burden. As discussed above, the reported levels of burden were significant and the results for the total mean score should be interpreted with caution. Concerning the personal strain and role strain dimensions of burden, 42% of caregivers noted high burden and 24% noted severe burden, emphasising the very real presence of burden among participants and giving weight to the arguments cited above. Knowing that the perception of poor health is also a predictor of perceived burden, it is important to reiterate that most of the relatives in our study reported a good perception of their overall health (61%). However, our main concern lies with the quarter of participants who considered their overall health to be average—a far from insignificant proportion. Family caregivers’ perceptions about their own health is a factor to be explored in future research about their care burden.

Being retired seemed to imply having a lower perceived burden than caregivers who had a job or were unemployed, probably because the retirees had more time available to provide informal care. However, only four caregivers scored below the median total ZBI score and were not retired, potentially biasing these results (*p* = 0.04).

The older adults with nervous system, osteoarticular, muscle, and connective tissue comorbidities raised their caregivers’ levels of burden to “moderate” compared to other active comorbidities that led to lower reported levels of burden. Variables associated with older adult patients appeared to have a greater influence on family caregivers’ perceived burden than variables associated with caregivers themselves [33]. Cognitive impairment seemed to be a central element in increasing a caregiver’s burden.

The caregiver’s gender showed no association with the average weekly time spent on informal caregiving and perception of burden, despite what has been reported in the literature [66]. This is probably related to the large disparity between the number of women and men in the sample. Our results tended to be consistent with some previous studies showing no statistically significant association between gender and perceptions of burden [31,32,66].

### 4.5. Associations between Preparedness for Caregiving and Perceptions of Burden

Although there has been no recent research investigating the associations between the dimensions of PCS and ZBI scores, the original PCS study had identified associations between role strain and levels of preparedness [48]. According to its authors, high levels of preparedness implied lower levels of role strain [48]. However, the constructs related to role strain used in the original study were not the same as those used by researchers working with the ZBI [14,15], which makes comparisons between their findings difficult. The present study’s results showed that the negative correlations between dimensions of the PCS and the ZBI were very low, particularly for correlations between each scale’s overall scores. Nevertheless, three correlations differed slightly: (a) PCS “Emotional support” and ZBI “Role strain”; (b) PCS “Organising in-home support services” and ZBI “Role strain”; and (c) PCS “Overall” and ZBI “Role strain”. These coefficients may indicate a potential association between these variables and future research should explore them. Despite the very low correlation coefficient values, an inverse association was found between measures of preparedness and burden, which is consistent with the findings of a previous study of caregivers of people with cancer [41]. However, due to the heterogeneity of the studied populations, these results should be interpreted with caution.

### 4.6. Strengths and Limitations

Recruiting caregivers to our study was particularly complex. Many patients refused to participate, limiting access to potentially vulnerable family caregivers (selection bias cannot be excluded). Despite the reminders issued, a considerable number of questionnaires were not returned. For reasons of feasibility, recruitment stopped at 38 “older adult–family caregiver” dyads. Moreover, male caregivers were under-represented in the sample, even if this outcome is consistent with previous studies. Potential temporal and confounding factors should also be considered, because caregivers are differently exposed to the factors causing feelings of burden, and some of them may be the informal caregiver for more than one older adult. Furthermore, family caregivers’ mental health (e.g., symptoms of depression or anxiety) could have been linked to their perceived levels of burden, but this specific data was not collected via the questionnaires administered, which limited further analysis of potential confounding variables. Finally, in terms of external validity, the potential to generalise these results is limited by their non-probabilistic sampling, a relatively small sample size, and the realities of a very localised setting.

Despite its limitations and to the best of our knowledge, this study is the first to simultaneously investigate the concepts of burden and preparedness for caregiving, and the associations between their different dimensions, in a French-speaking Swiss population. This study has contributed to documenting the caregiving role’s impact on the daily life of partially-dependent older adults, and to describing phenomena of interest to geriatric nursing care. Indeed, the initial research question was raised by geriatrics nurses as a direct result of their clinical practice, something which can support knowledge transfer and promote innovative clinical practices. Using validated measurement instruments previously used with similar populations was also one of the study’s strengths, and contributed to a better understanding of the family caregivers whom nurses meet in their daily practice.

### 4.7. Recommendations for Clinical Practice, Research, and Education

Our initial literature review highlighted that family caregivers’ burden is a well-documented reality internationally, with a negative impact on their QoL. Caregivers’ self-evaluation of their perceived preparedness for caregiving also appeared to be an important factor to consider, given preparedness’ association with mental health, anxiety, and burden. By focusing on family caregivers’ perceptions and using a systemic approach, the present study’s results should contribute to a better understanding of the realities faced by hospitalised older adults. Recommendations for clinical practice are based on a systemic, systematic, structured, and standardised assessment of the perceptions of burden and preparedness for caregiving. Based on these measures, specific nursing interventions that reinforce available resources and maintain the balance and QoL of family systems should be developed. Interventions should be linked to interdisciplinary collaboration, support, and education.

Longitudinal designs would allow future research on the evolution of these two phenomena over time. It would seem important to consider creating, optimizing, or testing educational programmes and counselling sessions for the caregivers of older adults whose functional independence is deteriorating. Given the small number of studies that have linked the concept of preparedness to the concept of burden, further research is needed, particularly in community settings and with larger samples. Future research should incorporate correlational analyses between the two main variables and older adults’ diagnoses and comorbidities, and it should investigate caregivers’ perceptions of their health status and whether those perceptions impact their QoL.

At the educational level, our recommendations would highlight the importance of training nurses to use a systemic family approach, so that they are well-prepared to provide good professional support and guide families towards internal and community resources. Nurses should also be trained in the use and interpretation of geriatrics screening tools, such as the PCS and the ZBI, which both have good psychometric properties. Thus, the integration of extra dimensions of assessment could help nurses to implement interventions which are more personalised and better adapted to the needs of older adults and their caregivers, and which helps to reach interdisciplinary care goals.

## 5. Conclusions

Although Western Switzerland’s home care services are well developed and older adults’ family caregivers claim to be satisfied with these services, the present study highlighted the existence of a significant perceived burden and low levels of preparedness for caregiving among this population. However, perceptions of burden and preparedness did not seem to be associated.

Given the negative impact of caregivers’ burden on their QoL and the associations between preparedness for caregiving, mental health, and anxiety, nurses should implement a systemic, systematic, structured, and standardized assessment of their clinical practice, including assessments of burden and preparedness for caregiving among family caregivers. The associations between burden and preparedness for caregiving in this population should be studied in a larger sample.

## Figures and Tables

**Table 1:** Family caregivers’ sociodemographic characteristics (*n* = 38).

Age, Mean (*SD*)	66.34 (16.44)
	*n* (%)
**Female**	30 (79.0)
**Nationality** (*n* = 37) *	
Swiss	28 (75.7)
Italian	3 (8.1)
Spanish	2 (5.4)
Other	4 (10.8)
**Marital status**	
Married	28 (73.7)
Single	6 (15.8)
Other	4 (10.5)
**Employment status**	
Full-time job	7 (18.4)
Part-time job	5 (13.2)
Retired	23 (60.5)
Jobless	3 (7.9)
**Monthly income (CHF)** (*n* = 35) *	
<2000	8 (22.9)
2000–5000	12 (34.3)
5001–7500	9 (25.7)
>7500	6 (17.2)
**Monthly income is a source of concern** (*n* = 37) *, *n* (%)	8 (21.6)
**Educational level**	
Elementary school	10 (26.3)
Apprenticeship or equivalent level	13 (34.2)
High school	2 (5.3)
University	13 (34.2)
**Family relationship with the older adult**	
Spouse	15 (39.5)
Child	15 (39.5)
Friend	4 (10.5)
Other	4 (10.5)
Cohabitation with the older adult	17 (44.7)
Household composition (*n* = 36) *	
One person	4 (11.1)
Two persons	27 (75.0)
≥ Three persons	5 (13.9)
**Urban residential area**	28 (73.7)
**Professional home care services for the family** **caregivers themselves** (*n* = 37) *	
Yes	4 (10.8)
Assistance with bathing	1 (25.0)
Assistance with housekeeping	2 (50.0)
Assistance with bathing and meal delivery	1 (25.0)
Frequency of the assistance	
Once a week	3 (75.0)
Three times a week	1 (25.0)
No	33 (89.2)

* *n* ≠ 38 due to nonrespondents. Abbreviations: *SD*—standard deviation; CHF—Swiss francs.

**Table 2:** The type of assistance provided by family caregivers before their older relative’s hospitalisation (*n* = 38).

Type of Assistance	*n* (%)
Assistance with bathing	13 (34.2)
Assistance with mobility and transfers	15 (39.5)
Assistance with dressing and undressing	11 (29.0)
Assistance with toileting	5 (13.2)
Assistance with feeding	16 (42.1)
Assistance with grocery shopping	30 (79.0)
Assistance with transportation	20 (52.6)
Assistance with medication	15 (39.5)
Assistance with housekeeping	17 (44.7)
Assistance with finances	24 (63.2)
Assistance with food preparation	25 (65.8)
Assistance with laundry	23 (60.5)
Occasional surveillance	18 (47.4)
Constant surveillance (day and night)	6 (15.8)

**Table 3:** Family caregivers’ perceptions of their preparedness for caregiving (*n* = 38).

Overall Level of Preparedness for Caregiving: Total Score (0–4)	Mean (*SD*)
	1.76 (0.91)
	*n* (%)
(0) Not at all prepared	1 (2.6)
(1) Not too well prepared	16 (42.1)
(2) Somewhat well prepared	14 (36.8)
(3) Quite well prepared	5 (13.2)
(4) Very well prepared	2 (5.3)

**Table 4:** Associations between the dimensions of *Preparedness for Caregiving* and the dimensions of *Burden* according to Spearman’s correlation coefficient (*ρ*).

Dimensions	ZBI Personal Strain		ZBI Role Strain		ZBI Overall	
	*ρ*	*p*-Value	*ρ*	*p*-Value	*ρ*	*p*-Value
**PCS** **Physical care**	−0.06	0.726	−0.05	0.780	−0.10	0.546
**PCS** **Emotional support**	−0.18	0.289	−0.35 *	0.034	−0.31	0.062
**PCS** **Organising in-home support services**	−0.23	0.172	−0.33 *	0.047	−0.23	0.164
**PCS** **Stress management**	−0.18	0.273	−0.23	0.174	−0.19	0.256
**PCS** **Overall**	−0.28	0.093	−0.33 *	0.041	−0.30	0.067

* Correlations are significant at the 0.05 level (*p* < 0.05). Abbreviations: ZBI—Zarit Burden Interview; PCS—Preparedness for Caregiving Scale.

## Data Availability

The data presented in this study are not publicly available due to ethical restrictions.
